# Insights into axial spondyloarthritis features and the development of imaging evidence of sacroiliitis during long-term follow-up in routine clinical practice

**DOI:** 10.1186/s13075-026-03811-z

**Published:** 2026-04-22

**Authors:** Stan Corné Kieskamp, Marjan Jolanda Nijmeijer, Fréke Wink, Reinhard Bos, Suzanne Arends, Anneke Spoorenberg

**Affiliations:** 1 Rheumatology and Clinical Immunology, University Medical Center Groningen, Hanzeplein 1, Groningen, 9713 GZ The Netherlands; 2 University of Groningen, Broerstraat 5, Groningen, 9712 CP The Netherlands; 3 Frisius Medical Center Leeuwarden, Henri Dunantweg 2, Rheumatology, Leeuwarden, 8934 AD The Netherlands

## Abstract

**Objective:**

To analyse characteristics and SpA features of newly diagnosed patients with radiographic (r-) versus non-radiographic (nr-)axSpA, and nr-axSpA patients fulfilling the imaging versus clinical arm of the ASAS classification criteria. Furthermore, this study explored whether patients from the clinical arm developed imaging evidence of sacroiliitis during follow-up.

**Methods:**

Consecutive newly diagnosed axSpA patients included in the prospective GLAS cohort between 2009 and 2019 were analysed. Demographic data and SpA features were compared between groups using multivariable logistic regression analysis. Patients fulfilling the clinical arm were evaluated for the development of radiographic sacroiliitis during follow-up.

**Results:**

305 patients with a recent axSpA diagnosis were included. Mean age was 38 ± 12 years, 55% were males, median diagnostic delay was 6 (IQR 2–13) years, and 75% were HLA-B27+. Patients diagnosed with r-axSpA (*n* = 170; 56%) were more often male and had more often elevated CRP than patients diagnosed with nr-axSpA. Patients with nr-axSpA had more often inflammatory back pain and a positive family history of SpA. Clinical diagnosis was made without imaging evidence of sacroiliitis in 38 (12%) patients. Only 2 (5%) of these patients later developed sacroiliitis on imaging (median follow-up 8 years).

**Conclusion:**

Overall, diagnosed and classified axSpA patients have similar clinical presentation and disease burden. In approximately 1 in 10 patients, axSpA diagnosis was made without imaging evidence of sacroiliitis, with a negligible number developing sacroiliitis on imaging during long-term follow-up. Imaging may improve diagnostic confidence, which can prevent reconsideration of the diagnosis and reduce inappropriate management decisions and expectations.

## Introduction

Axial spondyloarthritis (axSpA) is a chronic rheumatic auto-inflammatory disease primarily affecting the axial skeleton. It is characterised by inflammation of the sacroiliac joints (sacroiliitis) and spine (spondylitis), resulting in symptoms of chronic (inflammatory) back pain (IBP) and stiffness [1]. Clinical diagnosis is made by the rheumatologist based on pattern recognition of clinical, imaging and laboratory SpA features. The presence of chronic back pain together with signs of sacroiliitis on imaging, in combination with the presence of human leukocyte antigen B27 (HLA-B27), the most important genetic factor [2], and with at least one other SpA feature makes the diagnosis likely [1]. For this, sacroiliitis on imaging can be identified either by demonstrating structural damage on pelvic X-ray, or signs of active sacroiliitis on sacroiliac magnetic resonance imaging (MRI) scan. In general, the likelihood of a SpA diagnosis increases with the number of SpA features present.

AxSpA can be categorised in two diagnostic phenotypes: radiographic axSpA (r-axSpA), characterised by sacroiliitis on pelvic radiograph, and non-radiographic axSpA (nr-axSpA). Clinically, patients with r-axSpA and nr-axSpA share comparable burdens of disease, treatment modalities and treatment effects. Patients with r-axSpA are more often male, and have more often inflammation, as shown by higher C-reactive protein (CRP) and more inflammatory sacroiliac (SI) lesions on MRI, compared to patients with nr-axSpA [3].

In 2009, the Assessment of SpondyloArthritis international Society (ASAS) classification criteria for axSpA were developed to facilitate a more homogenous selection of axSpA patients for research including clinical trials [4]. Patients with chronic low back pain with onset before the age of 45 years can be classified as axSpA based on two arms: (1) the imaging arm for which sacroiilitis on pelvic radiograph or MRI is mandatory in combination with at least one additional SpA feature and (2) the clinical arm for which the presence of HLA-B27 is mandatory with at least two additional SpA features. In the validation study, the specificity of the clinical arm in respect to the clinical diagnosis made by an expert physician was lower in comparison to the imaging arm, 85% and 97%, respectively. It should also be noted that the validation study was done in a tertiary axSpA expertise referral center, and therefore it can be expected that the specificity may be lower in daily rheumatology practice.

In daily clinical practice, rheumatologists experience difficulties in diagnosing axSpA, especially nr-axSpA, because they may lack the knowledge and experience to recognize SpA-related sacroiliitis on MRI and its associated pitfalls [5]. The risk of overdiagnosis may be higher when using classification criteria as diagnostic criteria in patients who fulfil the clinical arm of the ASAS criteria and show no sacroiliitis on imaging, which is considered the hallmark of axSpA. For example, an HLA-B27 positive patient with IBP and enthesitis can be classified as nr-axSpA. However, this may be insufficient for a definite axSpA diagnosis, and maybe even another SpA phenotype should be considered, as IBP is not a very specific feature for axSpA [6].

Misdiagnosis can lead to inappropriate treatment and management decisions and expectations. Knowledge of the characteristics and features of newly diagnosed axSpA patients will help to better recognize and diagnose axSpA, especially nr-axSpA [7]. Therefore, in our large, long-term cohort of axSpA patients from routine clinical practice our first aim was to analyse characteristics and SpA features of newly diagnosed axSpA patients stratified by the clinical diagnosis r-axSpA and nr-axSpA, and nr-axSpA patients stratified by fulfilment of the imaging- and the clinical arm of the ASAS classification criteria. Our second aim was to investigate whether nr-axSpA patients who fulfil the clinical arm of the classification criteria develop imaging evidence of sacroiliitis during long-term follow-up in routine clinical practice.

## Patients and methods

This study is a cross-sectional analysis at baseline with additional longitudinal follow-up for imaging progression of consecutive axSpA patients diagnosed shortly (≤ 1 year) before inclusion in the Groningen Leeuwarden Axial Spondyloarthritis (GLAS) cohort between 2009 and 2019. The GLAS cohort is an ongoing prospective observational cohort study with follow-up visits according to a fixed protocol [8]. Since 2009, patients over 18 years of age, diagnosed with axSpA according to their treating rheumatologists and fulfilling the ASAS classification criteria [4] have been included, irrespective of treatment, in the Frisius Medical Center (FMC), a secondary referral center, and the University Medical Center Groningen (UMCG), a tertiary referral center. The GLAS cohort was approved by the local ethics committees of the MCL and the UMCG (RTPO 364/604). All participants provided written informed consent according to the Declaration of Helsinki.

### Patient characteristics and SpA features

Patient characteristics at baseline were age, sex, BMI, current smoking status, age at symptom onset, and diagnostic delay. In addition, the following SpA features were collected at baseline: IBP according to the ASAS criteria; peripheral manifestations (peripheral arthritis, enthesitis and dactylitis); extraskeletal manifestations (ESMs; uveitis, psoriasis, IBD); patient-reported good response to NSAIDs; family history of SpA; and HLA-B27 status. Disease activity assessments used were Axial Spondyloarthritis Disease Activity Score (ASDAS) and the validated cut-off value for high disease activity (ASDAS ≥ 2.1); C-reactive protein (CRP) and elevated CRP level (≥ 5 mg/L); and Bath Ankylosing Spondylitis Disease Activity Index (BASDAI) and the validated cut-off value for active disease (BASDAI ≥ 4). Regarding imaging, sacroiliitis was assessed on pelvic radiograph according to the modified New York (mNY) criteria [9] by their treating rheumatologist. Sacroiliitis was assessed on MRI according to the ASAS criteria [10] by axSpA clinical expert rheumatologists (AS and FW) and when in doubt, in consensus with an experienced musculoskeletal radiologist of a tertiary referral center. Readers were not blinded for clinical data according to routine clinical practice. In general, MRI of the SI joints with short tau inversion recovery (STIR) images and T1-weighted sequences were evaluated for both active inflammatory lesions (primarily bone marrow oedema) and structural lesions (e.g. erosions, sclerosis and fat infiltration). MRI at baseline was not mandatory as part of the study protocol. Therefore, the decision to perform an MRI was left to the discretion of the rheumatologist. Radiographs of the pelvis, acquired before diagnosis and then every 2 years according to the standardised protocol of the cohort, as well as all available MRI’s acquired before the diagnosis and during follow-up, were evaluated to determine whether diagnosed axSpA patients fulfilling the clinical arm of the ASAS classification criteria developed imaging evidence of sacroiliitis. This was defined as grade ≥ 2 bilaterally or grade ≥ 3 unilaterally according to the mNY criteria (radiograph), or as either sacroiliitis according to the ASAS criteria or structural lesions associated with sacroiliitis (MRI), during follow-up.

### Statistical analysis

Patient characteristics and SpA features were presented as number of patients (%), mean ± standard deviation (SD), or median (interquartile range; IQR) for categorical, normally distributed, and non-normally distributed variables, respectively. Prevalence of the characteristics and SpA features were presented for the entire study population stratified by clinical diagnosis of r-axSpA versus nr-axSpA, and nr-axSpA patients stratified by fulfilment of the imaging- and the clinical arm of the ASAS classification criteria. Differences between groups were compared using Chi-Square test or Fisher’s Exact test, Independent Samples t-test or Mann-Whitney U test as appropriate for data type and distribution. Additionally, univariable and multivariable logistic regressions were used to explore the association of SpA features with the clinical diagnosis or arms of the classification criteria. Missing data were not imputed and in the multivariable analyses were managed through pair-wise deletion. The percentage of missing data for each parameter is indicated in the tables. Statistical analysis was performed using IBM SPSS Statistics 28.0.0. P-values of < 0.05 were considered statistically significant.

## Results

A total of 305 patients with a recent (≤ 1 year) clinical diagnosis of axSpA and fulfilling the ASAS classification criteria were included in this study. At baseline, their mean age was 38 (SD ± 12) years, 168 (55%) patients were male, median diagnostic delay was 6 years (IQR 2–13), 230 (75%) patients were HLA-B27 positive, 242 (82%) had a history of IBP, 159 (62%) experienced a good response to NSAIDs and 85 (29%) reported a positive family history for SpA. In total, 107 (36%) had a peripheral manifestation (current or in the past) and 98 (32%) were diagnosed with ≥ 1 ESM. Regarding disease activity, mean ASDAS was 2.7 (± 1.1) and 186 (70%) patients had active disease defined as ASDAS ≥ 2.1, median CRP was 3.1 (2.0–8.0) and 114 (37%) patients had elevated CRP (≥ 5 mg/L), mean BASDAI was 4.6 (± 2.2) and 180 (60%) patients experienced active disease defined as BASDAI ≥ 4. In total, 93 (30%) patients started treatment with a biological disease modifying antirheumatic drug (bDMARD) within 3 months after baseline GLAS visit (Table 1).

**Table 1:** Patient characteristics and SpA features stratified by patients diagnosed with r-axSpA and nr-axSpA

	All Patients (*n*=305)	*r*-axSpA (*n* = 170, 56%)	nr-axSpA (*n* = 135, 44%)	*P*-value
Patient characteristics				
Age at symptom onset (yrs)^1^	25 (19–34)	25 (19–33)	25 (19–36)	0.96
Diagnostic delay (yrs)^1^	6 (2-13)	8 (2–13)	5 (2–15)	0.17
Sex (male)	168 (55%)	111 (65%)	57 (42%)	< 0.001*
BMI (kg/m^2^)^1^	26.4 ± 5.0	26.7 ± 5.3	26.1 ± 4.7	0.32
Current smoking^1^	89 (31%)	59 (36%)	30 (24%)	0.039*
Starting bDMARD	93 (30%)	50 (29%)	43 (32%)	0.71
Disease activity				
ASDAS^1^	2.7 ± 1.1	2.8 ± 1.1	2.6 ± 1.0	0.11
ASDAS ≥ 2.1	186 (70%)	106 (70%)	80 (70%)	1.00
BASDAI^1^	4.6 ± 2.2	4.6 ± 2.4	4.5 ± 2.1	0.64
BASDAI ≥ 4	180 (60%)	103 (62%)	77 (58%)	0.63
ASAS SpA features				
History of IBP^1^	242 (82%)	124 (77%)	118 (89%)	0.009*
History of peripheral manifestations	107 (36%)	58 (35%)	49 (37%)	0.72
Peripheral arthritis	74 (24%)	42 (25%)	32 (24%)	0.89
Enthesitis^1^	39 (13%)	18 (11%)	21 (16%)	0.23
Dactylitis^1^	17 (6%)	13 (8%)	4 (3%)	0.082
History of ESM	98 (32%)	56 (33%)	42 (31%)	0.81
History of uveitis	48 (16%)	32 (19%)	16 (12%)	0.11
History of psoriasis	41 (14%)	18 (11%)	23 (17%)	0.13
History of IBD	27 (9%)	19 (11%)	8 (6%)	0.15
Good response to NSAID^2^	159 (62%)	88 (64%)	71 (59%)	0.44
Family history for SpA^1^	85 (29%)	38 (23%)	47 (35%)	0.021*
HLA-B27	230 (75%)	130 (77%)	100 (74%)	0.69
CRP (mg/L)^1^	3.1 (2.0.8.0)	4.9 (2.0–10.0)	2.1 (2.0–6.0)	< 0.001*
Elevated CRP	114 (37%)	78 (46%)	36 (27%)	< 0.001*
Number of ASAS classification criteria fulfilled^3^	3.7 ± 1.4	3.7 ± 1.5	3.7 ± 1.3	0.78

Values are presented as n (%), mean ± SD or median (interquartile range). *p < 0.05. ^1^1-5% missing; ^2^15% missing; ^3^20% missing

*R-axSpA* radiographic axial spondyloarthritis, *nr-axSpA* non-radiographic axial spondyloarthritis, *bDMARD* biological disease-modifying antirheumatic drug, *ASDAS* Axial Spondyloarthritis Disease Activity Score, *BASDAI* Bath Ankylosing Spondylitis Disease Activity Index, *IBP* inflammatory back pain, *peripheral manifestation* peripheral arthritis, enthesitis and/or dactylitis, *ESM* extraskeletal manifestation, uveitis, psoriasis and/or IBD, *IBD* inflammatory bowel disease, *NSAID* nonsteroidal anti-inflammatory drug, *HLA-B27* human leukocyte antigen B27, *CRP* C-reactive proteins, *elevated CRP* CRP > 5, *ASAS* Assessment of SpondyloArthritis international Society

### Clinical diagnosis: r-axSpA versus nr-axSpA

Of the 305 axSpA patients, 170 (56%) were clinically diagnosed with r-axSpA and 135 (44%) with nr-axSpA. R-axSpA patients were significantly more often male (65% vs. 42%, *p* < 0.001) compared to patients diagnosed with nr-axSpA, and were more often current smokers (36% vs. 24%, *p* = 0.039). Diagnostic delay and the disease activity assessments ASDAS and BASDAI were not significantly different between patients diagnosed with r-axSpA or nr-axSpA.

Regarding the presence of SpA features of the ASAS classification criteria, r-axSpA patients had more often elevated CRP (46% vs. 27%, *p* < 0.001) and higher CRP (median 4.9 mg/L (2.0–10.0) vs. 2.1 mg/L (2.0–6.0), *p* < 0.001) than patients with nr-axSpA. Patients diagnosed with nr-axSpA had significantly more often IBP (89% vs. 77%, *p* = 0.009) and a positive family history for SpA (35% vs. 23%, *p* = 0.028) than patients with r-axSpA. All other patient characteristics and SpA features were not significantly different between the two groups. Also, the total number of SpA features was not significantly different between r-axSpA and nr-axSpA patients (mean 3.7 vs. 3.7, *p* = 0.78) (Table 1). Multivariable logistic regression showed that the SpA features history of IBP (OR 0.26) and family history for SpA (OR 0.52) were negatively associated with the clinical diagnosis of r-axSpA, whereas an elevated CRP (OR 1.91) was positively associated with the clinical diagnosis of r-axSpA, with a combined explained variance of 12.6% (Table 2).

**Table 2:** Logistic regression analysis of SpA features associated with clinical diagnosis of r-axSpA vs. nr-axSpA

	Univariable model	Multivariable model
	OR (95% CI)	OR (95% CI)
History of IBP	0.43 (0.22–0.82)*	0.26 (0.11–0.63)**
History of peripheral arthritis	1.05 (0.62–1.77)	
History of enthesitis	0.65 (0.33–1.27)	
History of dactylitis	2.76 (0.88–8.68)	
History of uveitis	1.74 (0.91–3.32)	
History of psoriasis	0.57 (0.29–1.11)	
History of IBD	2.00 (0.85–4.72)	
Good response to NSAIDs	1.24 (0.75–2.05)	
Family history for SpA	0.55 (0.33–0.91)*	0.52 (0.29–0.94)*
HLA-B27+	1.14 (0.67–1.92)	
Elevated CRP	2.33 (1.43–3.79)***	1.91 (1.10–3.33)*

R^2^ for multivariable model equals 0.126. *p < 0.05, **p < 0.01, ***p < 0.001

*IBP* inflammatory back pain, *IBD* inflammatory bowel disease, *NSAID* nonsteroidal anti-inflammatory drug, *SpA* spondyloarthritis, *HLA-B27* human leukocyte antigen B27, *CRP* C-reactive protein

### nr-axSpA clinical diagnosis stratified by fulfilment of the imaging- or clinical arm of the ASAS classification criteria

Of the 135 diagnosed nr-axSpA patients, 97 (72%) fulfilled the imaging arm and 38 (28%; 12% of all axSpA patients) fulfilled the clinical arm of the ASAS classification criteria. Patient characteristics and disease activity were not significantly different (Table 3).

**Table 3:** Patient characteristics and SpA features of patients diagnosed with nr-axSpA stratified by the imaging and the clinical arm of the ASAS classification criteria

	Nr-axSpA (*n* = 135)	Nr-axSpA imaging arm (*n* = 97, 72%)	Nr-axSpA clinical arm (*n* = 38, 28%)	*P*-value
Patient characteristics				
Age at symptom onset (yrs)^1^	25 (19–36)	26 (19–36)	22 (17–34)	0.15
Diagnostic delay (yrs)^1^	5 (2–15)	4 (2–15)	6 (1–17)	0.33
Sex (male)	57 (42%)	42 (43%)	15 (39%)	0.70
BMI (kg/m^2^)^1^	26.1 ± 4.7	26.0 ± 5.0	26.2 ± 4.0	0.74
Current smoking^2^	30 (24%)	21 (24%)	9 (25%)	1.00
Starting bDMARD	43 (32%)	35 (36%)	8 (21%)	0.15
Disease activity				
ASDAS^1^	2.6 ± 1.0	2.6 ± 1.0	2.6 ± 1.1	0.94
ASDAS ≥ 2.1	80 (70%)	60 (71%)	20 (69%)	0.65
BASDAI^1^	4.5 ± 2.1	4.5 ± 2.1	4.6 ± 2.0	0.84
BASDAI ≥ 4	77 (58%)	53 (58%)	23 (61%)	0.85
ASAS SpA features				
History of IBP^1^	118 (89%)	84 (88%)	34 (92%)	0.56
History of peripheral manifestations	49 (37%)	37 (39%)	12 (32%)	0.55
Peripheral arthritis	32 (24%)	26 (27%)	6 (16%)	0.19
Enthesitis^1^	21 (16%)	15 (16%)	6 (16%)	1.00
Dactylitis	4 (3%)	4 (4%)	0 (0%)	0.58
History of ESM	42 (31%)	28 (29%)	14 (37%)	0.41
History of uveitis	16 (12%)	6 (6%)	10 (26%)	0.002*
History of psoriasis^1^	23 (17%)	19 (20%)	4 (11%)	0.31
History of IBD	8 (6%)	7 (7%)	1 (3%)	0.44
Good response to NSAID^3^	71 (59%)	47 (55%)	24 (69%)	0.22
Family history for SpA^1^	47 (35%)	28 (29%)	19 (50%)	0.029*
HLA-B27	100 (74%)	62 (64%)	38 (100%)	< 0.001*
CRP (mg/L)^1^	2.1 (2.0–6.0)	2.1 (2.0-6.5)	2.0 (2.0-4.8)	0.85
Elevated CRP	36 (27%)	28 (29%)	8 (21%)	0.40
Number of ASAS classification criteria fulfilled^4^	3.7 ± 1.3	3.5 ± 1.4	4.0 ± 1.0	0.042*

Values are presented as n (%), mean ± SD or median (interquartile range). *p < 0.05. ^1^1-5% missing; ^2^7% missing; ^3^11% missing; ^4^16% missing

*R-axSpA* radiographic axial spondyloarthritis, *nr-axSpA* non-radiographic axial spondyloarthritis, *bDMARD* biological disease-modifying antirheumatic drug, *ASDAS* Axial Spondyloarthritis Disease Activity Score, *BASDAI* Bath Ankylosing Spondylitis Disease Activity Index, *IBP* inflammatory back pain, *peripheral manifestation* peripheral arthritis, enthesitis and/or dactylitis, *ESM* extraskeletal manifestation, uveitis, psoriasis and/or IBD, *IBD* inflammatory bowel disease, *NSAID* nonsteroidal anti-inflammatory drug, *HLA-B27* human leukocyte antigen B27, *CRP* C-reactive proteins, *elevated CRP* CRP > 5, *ASAS* Assessment of SpondyloArthritis international Society

Regarding the presence of SpA features in patients with nr-axSpA only, patients fulfilling the clinical arm showed more often a history of uveitis (26% vs. 6%, *p* = 0.002), a positive family history for SpA (50% vs. 29%, *p* = 0.029) and had overall more SpA features compared to patients fulfilling the imaging arm (mean 4.0 vs. 3.5, *p* = 0.042) (Table 2). Multivariable logistic regression showed that only having a history of uveitis (OR 4.96) was independently associated with classification in the clinical arm of the classification criteria in nr-axSpA patients, with an explained variance of 9.5% (Table 4).

**Table 4:** Logistic regression analysis of SpA features associated with fulfilling the clinical vs. imaging arm of the ASAS classification criteria in patients with nr-axSpA

	Univariable model	Multivariable model
	OR (95% CI)	OR (95% CI)
History of IBP	1.62 (0.43–6.10)	
History of peripheral arthritis	0.50 (0.19–1.35)	
History of enthesitis	1.05 (0.37–2.94)	
History of dactylitis	N/A	
History of uveitis	5.42 (1.81–16.23)**	4.96 (1.60–15.40)**
History of psoriasis	0.49 (0.16–1.56)	
History of IBD	0.35 (0.04–2.92)	
Good response to NSAIDs	1.76 (0.77–4.05)	
Family history for SpA	2.39 (1.10–5.19)*	
HLA-B27+	N/A^1^	
Elevated CRP	0.66 (0.27–1.61)	

R^2^ for multivariable model equals 0.095. *p < 0.05, **p < 0.01

*IBP* inflammatory back pain, *IBD* inflammatory bowel disease, *NSAID* nonsteroidal anti-inflammatory drug, *SpA* spondyloarthritis, *HLA-B27* human leukocyte antigen B27, *CRP* C-reactive protein. ^1^All patients in the clinical arm of the ASAS classification criteria are per definition HLA-B27 positive

In 20 of the 38 patients with nr-axSpA fulfilling the clinical arm of the classification criteria, an MRI scan was performed at baseline and neither inflammatory lesions nor structural lesions associated with sacroiliitis were observed. In the remaining 18 patients, an MRI of the SI joints was not performed. There were no significant differences in patient characteristics and SpA features of nr-axSpA patients with or without an MRI scan performed.

### Development of sacroiliitis in nr-axSpA patients fulfilling the clinical arm of the ASAS classification criteria

All 38 nr-axSpA patients fulfilling the clinical arm had 1 or more follow-up pelvic radiographs with a median follow-up time of 8 (IQR 4–11) years. In 36 patients (95% of clinical arm at baseline; 12% of all axSpA patients), plain pelvic radiographs showed no development of radiographic sacroiliitis during follow-up. Only 2 (5%) patients developed radiographic sacroiliitis according to the mNY criteria after 2 and 6 years of follow-up, respectively.

Eleven patients (29%) also received an MRI during follow-up, of which 9 patients also had an MRI at baseline without signs of sacroiliitis. Median follow-up time of the second MRI was 4 years. In two of these eleven patients, follow-up MRI showed both bone marrow oedema as well as structural lesions consistent with sacroiliitis; these were the same patients as those who developed radiographic sacroiliitis on plain radiograph. The follow-up MRI of the other 9 patients demonstrated no abnormalities – either bone marrow oedema or structural lesions – consistent with sacroiliitis.

## Discussion

Our cross-sectional study in a large cohort of newly diagnosed axSpA patients from routine clinical practice from a secondary and tertiary referral center shows many similarities in characteristics and SpA features in patients stratified by clinical diagnosis (r- or nr-axSpA) and nr-axSpA patients stratified by the two arms of the ASAS classification criteria (imaging or clinical). IBP and a positive family history were significantly more often present in nr-axSpA, whereas male sex, current smoking, and elevated CRP were more often present in r-axSpA. These data are in accordance with data from previous studies.

The higher prevalence of male sex, active smoking status and elevated CRP among patients with r-axSpA compared to those with nr-axSpA is in accordance with results from a meta-analysis [11]. In previous research, male sex was identified as a prognostic factor for more severe radiographic damage in r-axSpA patients [12]. Furthermore, smoking at baseline was predictive of spinal radiographic progression [13, 14], and elevated CRP at baseline was identified as a substantial predictor of radiographic sacroiliitis progression in patients from the GESPIC cohort [15, 16]. However, it is important to note that our multivariable model predicting clinical diagnosis or classification had a relatively low explained variance, indicating that other (unmeasured) parameters also play a role in the diagnostic process.

In our cohort, patients with r-axSpA showed IBP less often compared to patients with nr-axSpA (77% vs. 89%). These findings are in contrast to data from a previous meta-analysis, which showed an equal prevalence of IBP among r-axSpA and nr-axSpA patients (86% vs. 88%) [11]. A potential explanation may be that in our study only current IBP was used in the analyses, while patients with r-axSpA may have experienced IBP more often in the past and not currently.

By definition, patients fulfilling the clinical arm of the ASAS classification criteria need an additional SpA feature compared to those fulfilling the imaging arm. Accordingly, we found a higher mean number of SpA features present in patients from the clinical arm in comparison to the imaging arm (4.0 vs. 3.5). Data from a tertiary referral center showed that the clinical value and importance of different SpA features for detecting axSpA in patients with chronic low backpain < 45 years of age varies largely [17]. Sacroiliitis on imaging, elevated CRP and HLA-B27 seem to have the highest diagnostic discriminating ability [18]. In our study, nr-axSpA patients in the clinical arm had more frequently a positive family history and uveitis compared to nr-axSpA patients in the imaging arm. These observations can be attributed to the 100% prevalence of HLA-B27, a mandatory feature in the clinical arm, as this is strongly associated with both of these features [19, 20].

Our results indicate that axSpA patients fulfilling the clinical arm of the ASAS classification criteria seem to still have an uncertain diagnosis over time and therefore an increased risk of misdiagnosis. In these patients, radiographic evidence of axSpA was not present at the time of diagnosis, and they also rarely develop radiographic evidence of sacroiliitis during long-term follow-up. In our cohort, 12% of patients did not have imaging evidence of sacroiliitis at baseline nor did most of them develop imaging evidence of sacroiliitis during an average follow-up of 8 years.

Misdiagnosis may occur in randomised clinical trials (RCTs), e.g. evaluating the effect of biological treatment in nr-axSpA, in which patient inclusion is primarily based on the ASAS classification criteria where MRI with distinct inflammatory lesions based on active sacroiliitis or elevated CRP must be present [21, 22]. This means that imaging evidence of active sacroiliitis is not mandatory, and a patient could still be included in an RCT based on elevated CRP. In our cohort from routine clinical practice, no differences were found in the prevalence of peripheral arthritis between r-axSpA and nr-axSpA patients (25% vs. 24%), which is in line with the findings of a meta-analysis [23]. In recent RCTs, the number of nr-axSpA patients with peripheral arthritis was much higher, e.g. 41% in the BE MOBILE trials and 51% in the ABILITY-3 trial [21, 22]. These patients may have been included because they fulfilled the clinical arm of the ASAS classification criteria with elevated CRP, however their clinical diagnosis is possibly closer to peripheral or undifferentiated SpA than to axSpA.

To the best of our knowledge, this is the first study including patients who are clinically diagnosed with axSpA according to the treating rheumatologist and fulfil the ASAS classification criteria, which focuses on which ASAS SpA features are present in the imaging and clinical arms. Previous research has focused on specific aspects such as classification criteria, clinical presentation, or treatment responses, with limited comprehensive comparisons between patients clinically diagnosed with r-axSpA and nr-axSpA. One systematic literature review provided a comprehensive comparison of axSpA patients classified as r- or nr-axSpA concerning clinical presentation and disease burden [11], yet did not report the fulfilment of ASAS classification criteria or an extensive comparison between the imaging and clinical arms within nr-axSpA patients.

An important limitation of our study is that MRI was performed on clinical indication and therefore not in all patients with nr-axSpA; it was not part of our standardised protocol. Prospective studies including patients diagnosed with axSpA but without sacroiliitis on baseline MRI, with MRI as part of the follow-up protocol would provide important insights into this issue. Pelvic radiographs and MRIs were scored by rheumatologists as part of routine clinical practice and therefore they were not blinded to clinical data. This could have introduced confirmation bias, meaning false-positive scoring of imaging in patients clinically suspected of having axSpA. It should be taken into account that positive findings on MRI of the SI joints can also be present in asymptomatic healthy individuals, runners or women presenting with postpartum back pain [24]. Furthermore, pairwise deletion for missing data can cause bias if data are not missing completely at random. However, the impact in this study is likely to be minimal due to the low number of missing data.

Overall, the current analyses within this well-defined axSpA cohort reflect the diagnostic evaluation of nr-axSpA after the implementation of the ASAS classification criteria, along with its difficulties and pitfalls in routine clinical practice.

## Conclusion

Newly diagnosed patients with r-axSpA and nr-axSpA, as well as nr-axSpA patients fulfilling the imaging and clinical arms of the ASAS classification criteria, had similar disease burden and clinical presentation. Diagnosed r-axSpA patients were more often male and had higher CRP than patients with nr-axSpA. In nr-axSpA, a positive family history of SpA and presence of IBP seems to contribute more to the diagnosis than in r-axSpA. Clinical presentation of nr-axSpA patients fulfilling the clinical or imaging arm of the ASAS classification criteria is similar, and the few differences can be explained by the mandatory features for the two different arms. In approximately 1 out of 10 patients, axSpA diagnosis was made without imaging evidence of sacroiliitis, a finding that may reflect the entanglement of clinical diagnosis and use of the classification criteria in the nr-axSpA clinical arm. Notably, a negligible number of these patients developed imaging evidence of sacroiliitis over a median follow-up of 8 years. Accordingly, in these patients with chronic back pain it may be uncertain whether they really suffered from sacroiliitis. Sacroiliitis is considered the hallmark of axSpA, but in the absence of radiographic sacroiliitis there can be no definite proof of its presence without MRI. Making a diagnosis of axSpA has important consequences, both in treatment decisions (biological/targeted synthetic DMARDS) and in the psychological burden for the patient. It is currently debated whether imaging is mandatory for diagnosing axSpA. Some axSpA experts advocate for a diagnosis based on a strong clinical phenotype in combination with imaging evidence of sacroiliitis, while others express concerns that this approach may lead to underrecognition of patients with a strong clinical phenotype but negative or inconclusive imaging findings. Although further research is needed, these results provide additional insights into the value of MRI in routine clinical practice to enhance diagnostic confidence, particularly in challenging cases. Adhering to this practice may help to prevent reconsideration of the diagnosis and reduce inappropriate management decisions and expectations.

## Data Availability

The data that support the findings of this study are not openly available and are available from the corresponding author upon reasonable request.
